# Pattern of video game usage and video game disorder in Portugueses adolescents: A study about parental and peer attachment, parenting styles, and communication in parenting

**DOI:** 10.1192/j.eurpsy.2024.916

**Published:** 2024-08-27

**Authors:** A. M. Ribeiro, B. R. Maia

**Affiliations:** ^1^ Universidade Católica Portuguesa; ^2^Universidade Católica Portuguesa; Center for Philosophical and Humanistic Studies, Braga, Portugal

## Abstract

**Introduction:**

Video game disorder has been a subject of increasing interest, being associated with patterns of insecure attachment and authoritarian and permissive parenting styles. However, there is still a gap concerning the relationship between video game disorder and parent-child communication, one of the fundamental components of attachment to parents. Particularly in the Portuguese context, research on these topics and their interrelations is still scarce, thus remaining relatively unexplored.

**Objectives:**

To explore the pattern of video game usage and video game disorder, as well as their relationships with parental and peer attachment, parenting styles, and communication in parenting within a sample of Portuguese adolescents.

**Methods:**

150 Portuguese teenagers, recruited at public Portuguese schools, aged between 10 and 19 years old (mean age = 14.37, DP = 3.12; 52.7% girls (n = 79), and mostly living with both parents (79.7%, n = 106) fulfilled a sociodemographic and an academic questionnaire, a questionnaire on video game use patterns, the Video Game Disorder Scale - Short Version 9, the People in My Life Questionnaire, the Portuguese hetero-report version of the Parenting Styles and Dimensions Questionnaire: Short Versionand the Perception Scale of Parenting Communication.

**Results:**

The majority of the sample indicated 3rd childhood (n = 81, 54.0%), specifically at 8 years old, as the age of video game initiation and a playtime of less than or equal to 2 hours (n = 111, 74.0%), with only 2 participants found to have a video game disturbance index (1.3%). A positive association was found between video game disturbance and the average hours of gameplay, as well as a negative association with the age of game initiation. Additionally, relationships were explored, revealing that video game disturbance is negatively related to lower quality of attachment to parents and peers, positively related to an authoritative parenting style, and negatively related to less available, open, and affectionate parent-child communication.

**Conclusions:**

This study provides an in-depth understanding of adolescents’ behavior regarding video games, contributing to the knowledge of the topic in the Portuguese context. Furthermore, the identification of factors associated with video game disturbance allows for the development of remediation and prevention programs for this addictive disturbance, which are essential tools in psychological practice.

**Disclosure of Interest:**

None Declared

